# Growth and Dispersion Control of SnO_2_ Nanocrystals Employing an Amino Acid Ester Hydrochloride in Solution Synthesis: Microstructures and Photovoltaic Applications

**DOI:** 10.3390/ma16247649

**Published:** 2023-12-14

**Authors:** Nagisa Hattori, Saeid Vafaei, Ryoki Narita, Naohide Nagaya, Norimitsu Yoshida, Takashi Sugiura, Kazuhiro Manseki

**Affiliations:** 1 The Graduate School of Natural Science and Technology, Gifu University, Yanagido 1-1, Gifu 501-1193, Japanmanseki.kazuhiro.k4@f.gifu-u.ac.jp (K.M.); 2 Mechanical Engineering Department, Bradley University, 1501 West Bradley Avenue, Peoria, IL 61625, USA

**Keywords:** nanoparticles, sol-gel, SnO_2_, TEM, photovoltaics

## Abstract

Tin oxide (SnO_2_) is a technologically important semiconductor with versatile applications. In particular, attention is being paid to nanostructured SnO_2_ materials for use as a part of the constituents in perovskite solar cells (PSCs), an emerging renewable energy technology. This is mainly because SnO_2_ has high electron mobility, making it favorable for use in the electron transport layer (ETL) in these devices, in which SnO_2_ thin films play a role in extracting electrons from the adjacent light-absorber, i.e., lead halide perovskite compounds. Investigation of SnO_2_ solution synthesis under diverse reaction conditions is crucial in order to lay the foundation for the cost-effective production of PSCs. This research focuses on the facile catalyst-free synthesis of single-nanometer-scale SnO_2_ nanocrystals employing an aromatic organic ligand (as the structure-directing agent) and Sn(IV) salt in an aqueous solution. Most notably, the use of an aromatic amino acid ester hydrochloride salt—i.e., phenylalanine methyl ester hydrochloride (denoted as L hereafter)—allowed us to obtain an aqueous precursor solution containing a higher concentration of ligand L, in addition to facilitating the growth of SnO_2_ nanoparticles as small as 3 nm with a narrow size distribution, which were analyzed by means of high-resolution transmission electron microscopy (HR-TEM). Moreover, the nanoparticles were proved to be crystallized and uniformly dispersed in the reaction mixture. The environmentally benign, ethanol-based SnO_2_ nanofluids stabilized with the capping agent L for the Sn(IV) ions were also successfully obtained and spin-coated to produce a SnO_2_ nanoparticle film to serve as an ETL for PSCs. Several SnO_2_ ETLs that were created by varying the temperature of nanoparticle synthesis were examined to gain insight into the performance of PSCs. It is thought that reaction conditions that utilize high concentrations of ligand L to control the growth and dispersion of SnO_2_ nanoparticles could serve as useful criteria for designing SnO_2_ ETLs, since hydrochloride salt L can offer significant potential as a functional compound by controlling the microstructures of individual SnO_2_ nanoparticles and the self-assembly process to form nanostructured SnO_2_ thin films.

## 1. Introduction

Research on photofunctional metal oxides that can be applied to solar energy conversion processes has received increasing attention due to its potential for creating new technologies related to renewable energy, such as photovoltaics and the photocatalytic production of commodity chemicals. A large number of different semiconductor metal oxide nanoparticles and their thin films have been explored to date. One of the well-known approaches involves the production of mesoscopic thin-layered substrates consisting of titanium dioxide (TiO_2_), tin oxide (SnO_2_), and other transition metal oxide nanomaterials. These substrates have been designed for use in dye-sensitized and perovskite solar cells [1,2,3]. An in-depth understanding of the crystal growth of these semiconductor oxide nanoparticles is beneficial in more effectively manipulating the interface materials at the nanoscale to optimize device functionality. It is of particular importance to research these microstructures in their solid state—i.e., powder and film forms—to determine the size and morphology of the individual particles as well as their final products, specifically with respect to the surface structures and aggregation properties of these nanoparticles.

In recent years, there has been remarkable progress in research related to perovskite solar cells composed of metal oxide(s) and organic–inorganic lead-halide perovskite compounds (defined as ABX_3_, where A: organic ions, B: Pb ion, X: halogen ions) in both academia and industry [2]. In the case of miniature devices, while a power conversion efficiency (PCE) of over 25% has been achieved—which is about on par with that of conventional solar cells—it is evident that the PCE still has room for further improvement, especially for large-area solar panels. Consequently, research on the development of oxide nanoparticle thin films, which form the foundation for perovskite compounds, is considered to be important for opening up new perspectives in the development of low-cost PSCs. One of the major device structures uses mesoscopic-type nanoparticle thin films made through the deposition of colloidal metal oxide nanoparticles in solution. In this device, the metal oxide thin film(s) adjacent to the light-absorbing layer functions as the material for electron extraction and transport.

In regard to the preparation of nanoparticle thin films using non-vacuum-based approaches, there are two main strategies. The first one involves chemically synthesizing metal oxide nanoparticles in solution. This is followed by deposition using the dispersed solution of metal oxide nanoparticles on top of conductive substrates via various methods, such as spin coating and chemical bath deposition (CBD). The second strategy consists of solution deposition of a metal complex precursor by methods such as spin coating and electrochemical reactions. Both of these methods require an annealing process after deposition to stabilize the thin films.

SnO_2_ has been extensively studied for use as an electron transport layer (ETL) in PSCs because of its high electron mobility, chemical stability, and suitable bandgap (~3.6 eV) for combination with lead-halide perovskite compounds [4,5,6,7,8,9,10,11,12,13,14,15,16,17,18,19,20,21,22,23,24,25,26,27,28,29,30,31,32,33,34,35,36,37,38,39,40,41,42,43,44]. In terms of nanoparticle synthesis, it is most common for the growth of particles to be controlled via sol-gel and hydrothermal processes from suitable metal ion sources. Along with the technological development of PSCs, methods for controlling the sizes and shapes of SnO_2_ nanoparticles with dimensions of 10~20 nm and methods for forming nanostructured tin oxide thin films by making use of spin-coating and CBD processes have been reported along with the development of PSCs. However, there are still more design criteria for SnO_2_-based ETLs that should be further addressed, with a focus on the formation of nanoparticulates via solution synthesis, especially those with sizes as small as ~5 nm, in order to characterize the size-dependent performance of SnO_2_ ETLs in PSCs.

In our previous study, we demonstrated, using TEM images, that the hydrolysis and polycondensation of Sn(II) hydroxides led to the formation of aggregated SnO_2_ nanoparticles with crystallite sizes of 2~5 nm through a freeze-drying process in which Na_2_CO_3_ was used as a pH controller in an aqueous medium [45]. As the reactions of the tin species accelerated under these conditions, it is likely that such small nanoparticles with high surface energies agglomerated during the organic ligand (template)-free reaction. This aggregation hampered the preparation of thin films for device applications due to difficulties in dispersing the nanoparticles in the reaction solution.

In general, additives such as organic structure-directing agents (SDAs) or surfactants have routinely been utilized in order to fine tune the characteristics of nanoparticles, including their structures and dispersion properties. While amino acids are some of the common SDAs playing a major role in the chelation of metal–organic ligand structures to form a metal complex [46], aromatic amino acid derivatives tend to have lower solubility than other ligands due to the hydrophobicity of the phenyl moiety. We anticipate that the use of an amino acid ester hydrochloride as a ligand (SDA) will be advantageous for preparing a precursor solution in which the ligand concentrations can be varied greatly, resulting in the precise control of the size of SnO_2_ particles in the sub-10 nm range.

In this paper, we demonstrate the application of L-phenylalanine methyl ester hydrochloride—a commonly used compound in pharmaceutical, food, and bio-related research [47]—for effective SnO_2_ nanoparticle synthesis. In doing so, we hope that a new avenue for this compound’s utilization in electronic devices, specifically SnO_2_-based perovskite solar cells, is provided.

## 2. Materials and Methods

### 2.1. Chemicals

All chemicals were used as purchased. L-phenylalanine methyl ester hydrochloride (C_6_H_5_CH_2_CH(NH_2_)COOCH_3_·HCl) and tin(IV) chloride dihydrate (SnCl_4_·5H_2_O, >98.0%) were obtained from FUJIFILM Wako Pure Chemical Corporation. Ethanol (99.5%) was purchased from Kanto Chemical Co., Inc., Tokyo, Japan. H_2_O (resistivity: 18.2 MΩ·cm) was obtained from a Milli-Q^®^ integral water purification system (MERCK Ltd., Tokyo, Japan).

N,N-dimethylformamide (DMF, dehydrated, >99.5%), dimethyl sulfoxide (DMSO, dehydrated, 99.0%), 2-propanol (IPA, dehydrated, >99.7%), titanium isopropoxide (TTIP) (>97%), and acetonitrile (dehydrated, >99.5%) were purchased from KANTO CHEMICAL Co., Inc., Tokyo, Japan. Methylamine hydrobromide (MABr, >98.0%), cesium iodide (CsI, >99.0%), lead(II) iodide (PbI_2_, 99.99%), formamidine hydroiodide (FAI, 99.99%), lithium bis(trifluoromethanesulfonly)imide (Li-TFSI, >98.0%), and formamidine hydrobromide (FABr, 99.99%) were purchased from TOKYO CHEMICAL INDUSTRY CO., Ltd., Tokyo, Japan. Spiro-MeOTAD, chlorobenzene (CB, anhydrous, 99.8%) and 4-tert-butylpyridine (TBP, 98%) were obtained from SIGMA-ALDRICH, Co., St. Louis, MO, USA.

### 2.2. Synthesis of SnO_2_ Nanocrystals

In 40 mL of a solvent of water:ethanol = 1:1 (*v*/*v*), L-phenylalanine methyl ester hydrochloride (0.43 g) was first dissolved, and SnCl_4_·5H_2_O (0.77 g) was added. The solutions became cloudy after the addition of the Sn source. The mixture was then stirred at a constant temperature for 48 h, during which the reaction was carried out on a hot plate (set at 70 °C or 80 °C) while covering the sample container with aluminum foil. Except for a part of the TEM samples and solar cell samples, the reaction mixture was centrifuged at 8000 rpm for 5 min after the addition of water. Through this process, the organic ligand was removed. The solid residues were dried in air to obtain powder samples, which were used for XRD and Raman measurements.

### 2.3. Characterization of SnO_2_ Nanocrystals

SnO_2_ nanoparticles were characterized by using X-ray diffraction (XRD) (Rigaku RINT Ultima/PC with monochromated Cu–Kα radiation). The crystallite sizes of the SnO_2_ nanoparticles were calculated using the Scherrer equation (D = Kλ/βcosθ). D, K, λ, and θ correspond to the crystallite size, Scherrer constant (0.90), X-ray wavelength (1.54 Å), and Bragg angle, respectively. The visualization of the microstructures was performed by means of TEM (JEM-2100, Tokyo, Japan). A confocal Raman microscope, RAMANtouch (Nanophoton Corp., Tokyo, Japan), was used for the characterization of the SnO_2_ samples. Laser light of 532 nm was used to irradiate the powder samples, and the laser power was 10 mW/cm^2^. The diffuse reflectance spectra of the dried powder samples were obtained using a UV–Vis spectrophotometer (V-700, Jasco, Tokyo, Japan).

### 2.4. Preparation and Structural Characterization of SnO_2_ Thin Films

The reaction solution obtained via solution synthesis was dried in air, and to this residue, 10 mL of ethanol was added to form a colorless, transparent solution. The precursor solution was then spin-coated onto the SnO_2_ underlayer at 3000 rpm for 30 s and annealed for 5 min at 120 °C. SnO_2_ thin-film substrates were then coated with a TiO_2_ thin layer (non-doped compact TiO_2_) using a reported method [48] to suppress charge recombination at the surface of SnO_2_. A titanium isopropoxide (TTIP) solution was prepared by mixing 13 mL of ethanol, 0.34 mL of distilled water, 3 drops of concentrated nitric acid, and 1 mL of TTIP. This was spin-coated onto the SnO_2_ layer in two steps (step 1: 1500 rpm, 30 s; step 2: 1000 rpm, 60 s), followed by heat treatment at 500 °C for 30 min. The substrates were analyzed by means of scanning electron microscopy (SEM) (HITACHI S-4800).

### 2.5. Fabrication and Evaluation of Perovskite Solar Cells

UV-ozone treatment was performed after ETL deposition. A glove box was filled with nitrogen, where a humidity was maintained at approximately 20%. Prior to the following perovskite solution deposition, the coated FTO substrate was heated to 80 °C on a hot plate. The perovskite precursor solution was prepared in the same way as we previously reported [49]. MABr 0.14 mmol (0.0157 g), CsI 0.07 mmol (0.0182 g), FAI 1.19 mmol (0.204 g), and PbI_2_ 1.45 mmol (0.666 g) were dissolved in DMF 80 vol% (0.8 mL) and DMSO 20 vol% (0.2 mL) and stirred for 2 h at 80 °C on a hot plate. A perovskite layer with a composition of Cs_0.05_MA_0.1_FA_0.85_PbI_2.9_Br_0.1_·0.05PbI_2_ was formed. The substrate was then set in a spin coater, and 100 μL of the perovskite solution was dropped onto the substrate. Two-step coatings (1000 rpm for 10 s, 6000 rpm for 30 s) were carried out. A total of 25 s after the start of spin-coating, 200 μL of CB was dropped. The substrate was annealed on a hot plate at 100 °C for 1 h, and it was cooled naturally to room temperature.

In parallel, 0.12 mmol (0.0148 g) of FABr was added to 4 mL of IPA to prepare a solution (30 mM). Then, 100 μL of the FABr solution was coated onto the perovskite layer at 3000 rpm for 30 s. The substrate was heated on a hot plate at 80 °C. for 10 min. After heating, the substrate was allowed to cool to room temperature.

A total of 0.037 mmol (0.045 g) of a hole-transport material, Spiro-MeOTAD, was dissolved in 0.5 mL of CB. Separately, 0.18 mmol (0.052 g) of Li-TFSI was dissolved in 0.1 mL of acetonitrile. We added 10 μL of the acetonitrile solution and 17.75 μL of TBP to the Spiro-MeOTAD solution; 60 µL of the prepared solution was then spin-coated on the perovskite layer at 4000 rpm for 20 s. The substrate was left for 24 h in air. Finally, a gold layer was deposited on top of Spiro-MeOTAD using a thermal evaporation method.

The current–voltage (I-V) curves were obtained under simulated full sunlight (100 mW/cm^2^) at AM 1.5 using a solar simulator (Yamashita Denso, YSS-80A, Tokyo, Japan) in combination with a potentiostat (Hokuto Denko HSV-110) for analysis. The active area of the device was regulated at 0.09 cm^2^. The I-V curves were measured under reverse scanning conditions at a scan rate of 50 mV/s. A cross-sectional image of the device was obtained using scanning electron microscopy (SEM) (HITACHI S-4800).

## 3. Results and Discussion

With regard to SnO_2_ nanocrystals, our focus was on achieving size and dispersion control of single-nanometer-scale SnO_2_ nanoparticles that can enhance interconnectivity within thin films. Phenylalanine derivatives with the steric hindrance of the phenyl moiety are considered suitable for modifying SnO_2_ nanostructures. This is achieved through the effective suppression of the formation of large nanoparticles during solution synthesis. However, addressing solubility is crucial because it is well known that such an aromatic amino-acid compound is less soluble. We anticipated an improvement in solubility with the use of the ester ligand hydrochloride (L). Moreover, the use of a higher concentration of L provides dual positive effects, i.e., removing a liquid catalyst for hydrolysis and polycondensation and decreasing the size of SnO_2_ nanoparticles because of the following. It is thought that positively charged L, (C_6_H_5_)CH_2_CH(NH_3_^+^)COOCH_3_, forms in the acidic condition [50] and that the L molecules most likely bind to Sn(IV) ions via their oxygen atoms. Therefore, an increased concentration of L is necessary for the control of SnO_2_ nanoparticles’ growth and dispersion.

**Figure 1:**
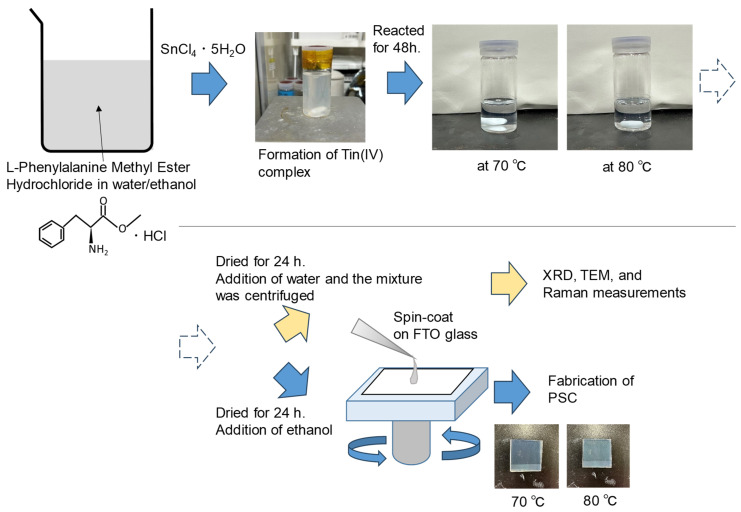
Schematics of SnO_2_ nanoparticle synthesis and the spin-coating deposition of the nanoparticles on an FTO substrate. An organic structure-directing agent (L-phenylalanine methyl ester hydrochloride) was added to a tin source to form the Sn(IV) complex, resulting in a cloudy reaction solution. The transparent, colorless solutions that resulted after hydrolysis and polycondensation were used to prepare the precursor solution for the spin-coating process.

**Figure 2:**
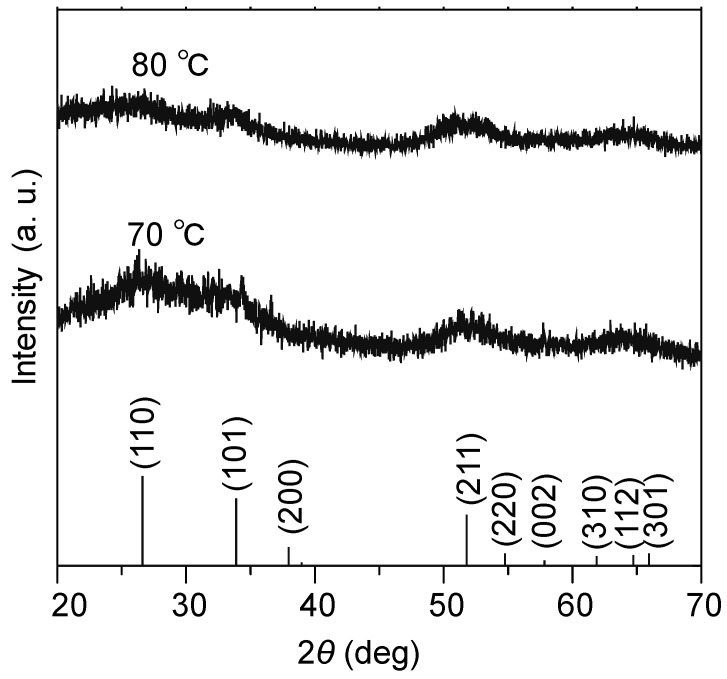
XRD pattern of the SnO_2_ nanoparticles synthesized at 70 °C to 80 °C. The bottom presents the database data of SnO_2_ (JCPDS: 00-041-1445).

As shown in Figure 1, we synthesized SnO_2_ nanocrystals by hydrolyzing Sn(IV) in the presence of phenylalanine methyl ester hydrochloride (L) in an aqueous solution of an ethanol/water mixture (for more details, refer to the Materials and Methods section). We were able to simultaneously control both crystal growth and dispersion at temperatures as low as 80 °C. As discussed below, it became evident that reaction temperatures ranging from 70 °C to 80 °C were critical conditions for optimizing the growth of well-dispersed SnO_2_ nanoparticles. From each reaction mixture at 70 °C and 80 °C, nanoparticles were isolated after drying in ambient air, and an XRD analysis was also conducted on them, as shown in Figure 2. In both samples, several broad peaks were observed, and all of them were assigned to those of the tetragonal SnO_2_ phases (PDF: 41-1445).

**Figure 3:**
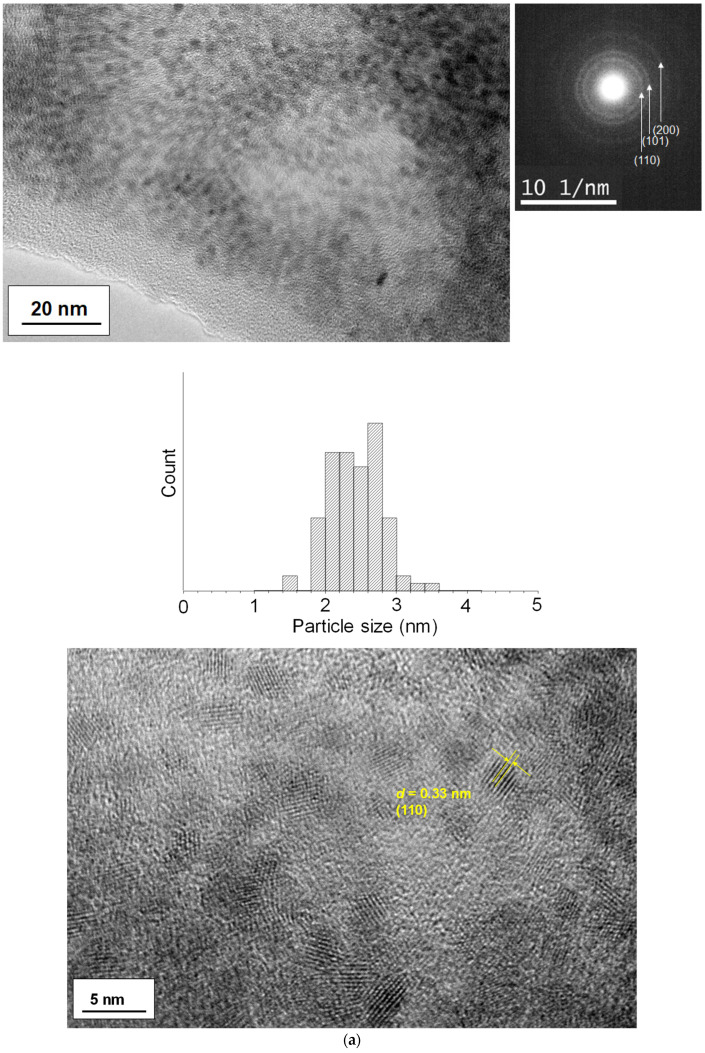

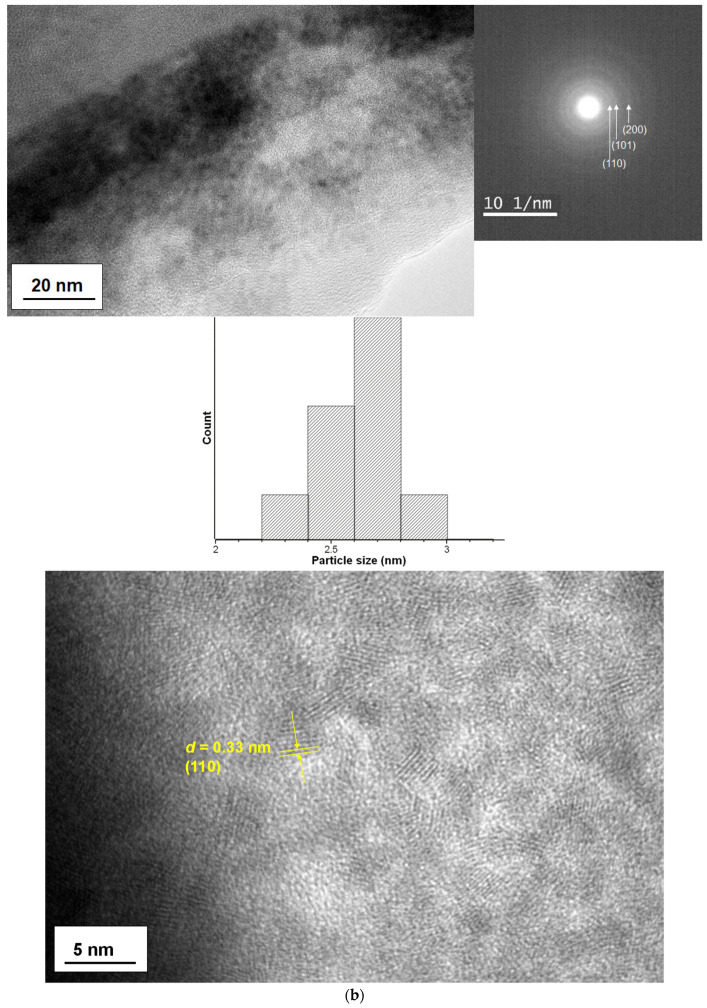
TEM images of as-synthesized SnO_2_ nanoparticles formed at (**a**) 70 °C and (**b**) 80 °C. The size distribution for each sample is also included. A *d*-spacing of the observed lattice fringes is presented in yellow.

We used transmission electron microscopy (TEM) to examine the dispersion of the SnO_2_ nanoparticles. Figure 3 displays data on the as-synthesized nanoparticles, including various magnified images and selected area diffraction (SAD) patterns. As seen in the sample at 70 °C (Figure 3a), well-dispersed single-nanometer-scale nanoparticles formed, with particle sizes of 2.4 ± 0.4 nm and a very narrow size distribution. When the reaction temperature was increased to 80 °C, the nanoparticles started to assemble, and the individual particle sizes (based on the lattice fringes) observed in the TEM images indicated a slight enhancement of particle growth. As for the 80 °C sample, the nanoparticles had sizes of 2.7 ± 0.2 nm. In both cases, the observed lattice fringes and SAD patterns from the high-resolution images were assigned to the SnO_2_ phase; the *d*-spacings were estimated to be 0.33 nm, corresponding to (110) planes, respectively. The crystallite sizes estimated using the Scherrer equation for (211) planes were 1.4 nm for 70 °C and 1.9 nm for 80 °C, respectively. Such a slight increase for the 80 °C sample is in good agreement with the observed particle sizes in the TEM images.

**Figure 4:**
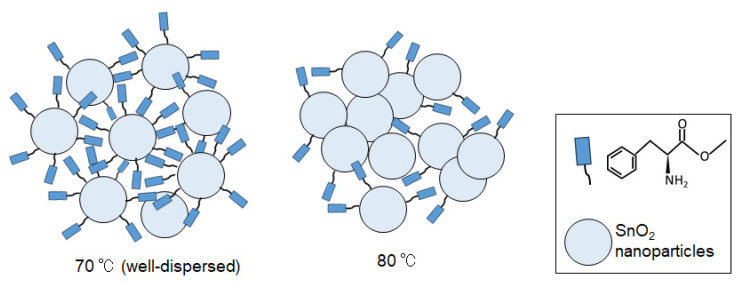
Conceptual drawings of the structures of SnO_2_ nanoparticles formed at 70 °C and 80 °C. They illustrate varying dispersion levels of nanoparticles, with the particle sizes in both conditions being approximately the same (2~3 nm).

As depicted in Figure 4, it is apparent from the data that the dispersion level of nanoparticles can be exclusively controlled using the reaction temperature. At 80 °C, it is thought that nanoparticle assembly was enhanced due to the removal of the organic ligands.

**Figure 5:**
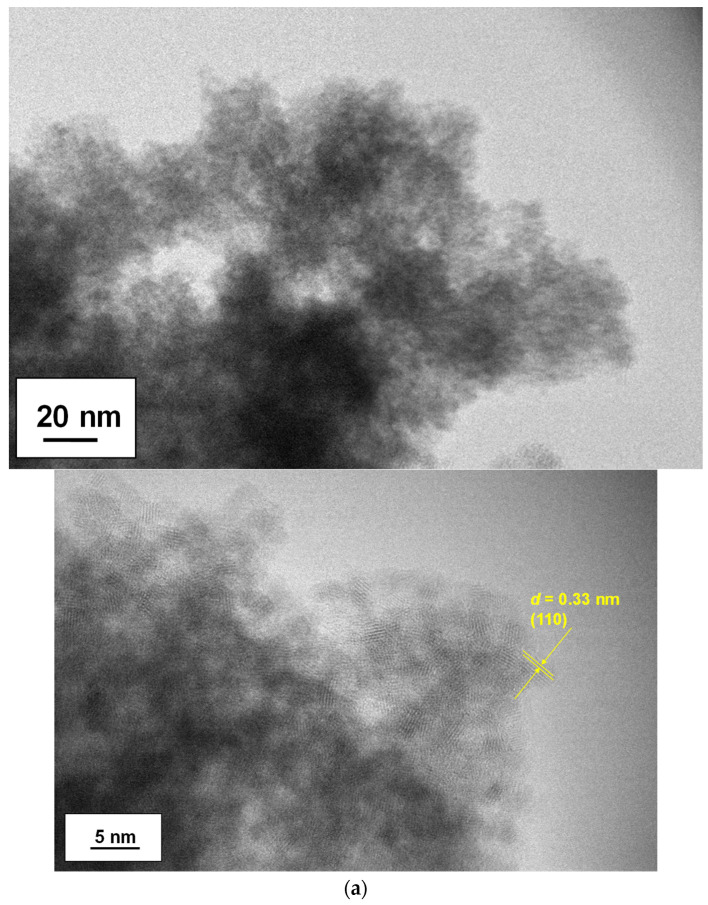

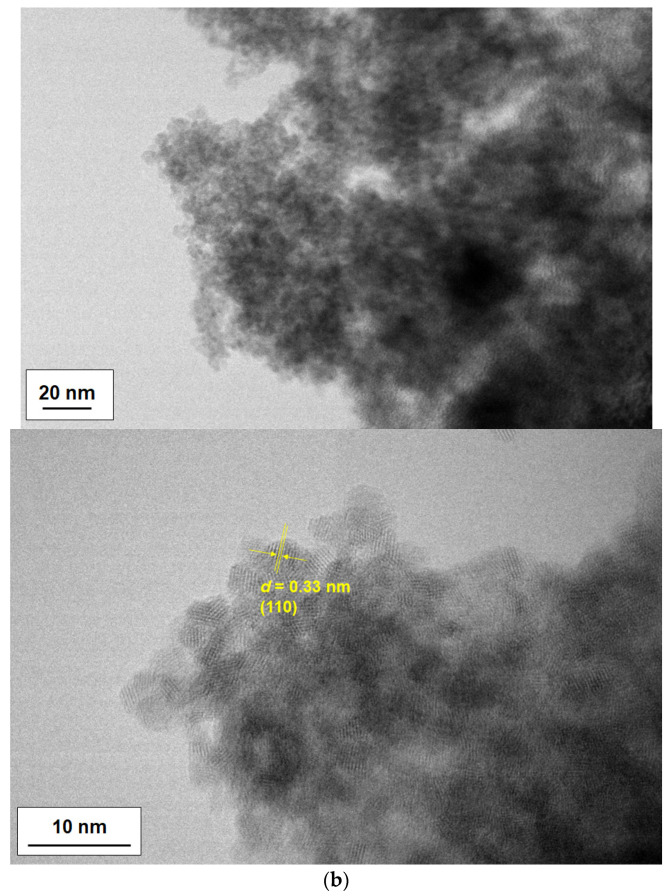
TEM images of SnO_2_ nanoparticles after a washing treatment. The synthesis of SnO_2_ nanoparticles was performed at (**a**) 70 °C and (**b**) 80 °C. A *d*-spacing of the observed lattice fringes is presented in yellow.

We also performed TEM (Figure 5) after a washing treatment with excess amount of water, which was expected to cause the complete removal of the capping ligands. The measurements revealed a significant aggregation of nanoparticles for both samples, while the particle sizes were maintained. Redispersion of the nanoparticles was not possible because of the aggregation of the particles, which hindered further applications.

There have been a few reports on the synthesis of SnO_2_ nanoparticles using an amino acid (not a HCl salt of an ester compound) as an SDA [51,52,53,54]. However, such a narrow size distribution and dispersion-controlled synthesis have not been demonstrated.

**Figure 6:**
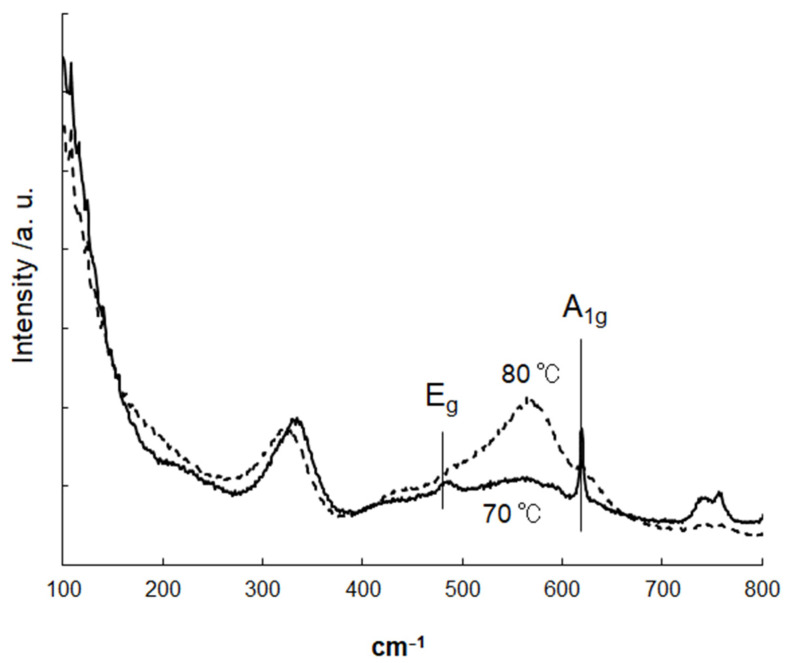
Raman spectra of SnO_2_ nanoparticles after a washing treatment. The synthesis of SnO_2_ nanoparticles was carried out at 70 °C and 80 °C.

The Raman spectra were also measured after the removal of organic ligands, as illustrated in Figure 6. Interestingly, two broad peaks were observed at approximately 330 cm^−1^ and 570 cm^−1^ for both 70 °C and 80 °C samples, which are not commonly seen in bulk single crystals or polycrystalline samples [55]. The 70 °C sample clearly displayed peaks at 476 cm^−1^ and 620 cm^−1^, which were assigned to vibration modes of E_g_ and A_1g_, the Raman active modes of rutile SnO_2_, respectively. Similar shoulder peaks at comparable locations were also identified in the 80 °C sample, likely originating from the same two bands. Liu et al. demonstrated that the existence of bridging oxygen vacancies downshifts the A_1g_ from ~635 cm^−1^ (bulk) to ~618 cm^−1^ [56]. These results suggested that the observed sharp peak at 620 cm^−1^ for the 70 °C sample arose from bridging oxygen vacancies in the SnO_2_ nanoparticles, whereas the corresponding peak intensity of the 80 °C sample was significantly decreased. Cheng et al. reported on the abovementioned Raman scattering peaks (observed at 330 cm^−1^ and 570 cm^−1^) and noted that they are sensitive to crystal surface area; these phonon modes are caused by small crystal size [57]. A broad, unidentified peak was observed in both SnO_2_ samples within the range of 400–450 cm^−1^. It is likely that not only the grain size but also local disorder may have influenced the Raman scattering, potentially resulting in the appearance of new modes in such a nanocrystalline sample [55].

**Figure 7:**
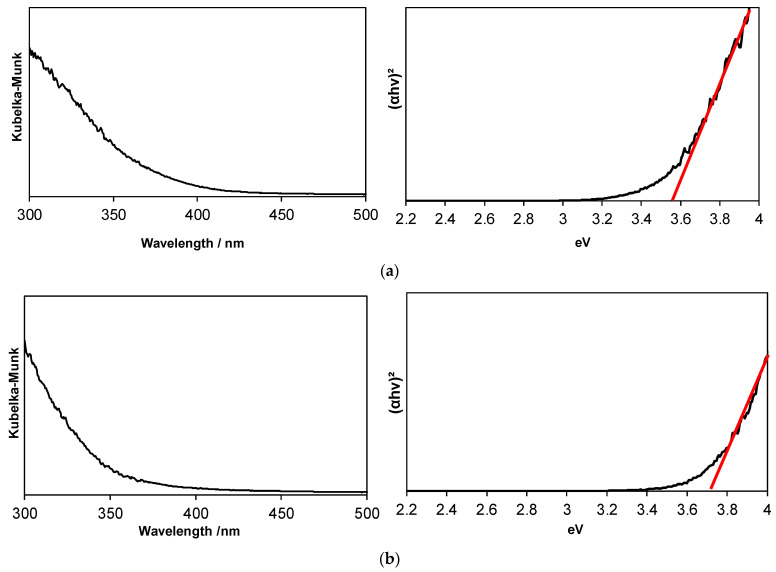
Photoabsorption spectra of SnO_2_ nanoparticles after a washing treatment. The synthesis reactions were performed at (**a**) 70 °C and (**b**) 80 °C. The linear part of each Tauc plot is extrapolated to the x-axis using the red line.

From the diffuse reflectance spectra, the band gaps of the powder samples were estimated to be 3.6 eV and 3.7 eV for the 70 °C and 80 °C samples, respectively (see Figure 7). These values are consistent with previously reported data [58].

**Figure 8:**
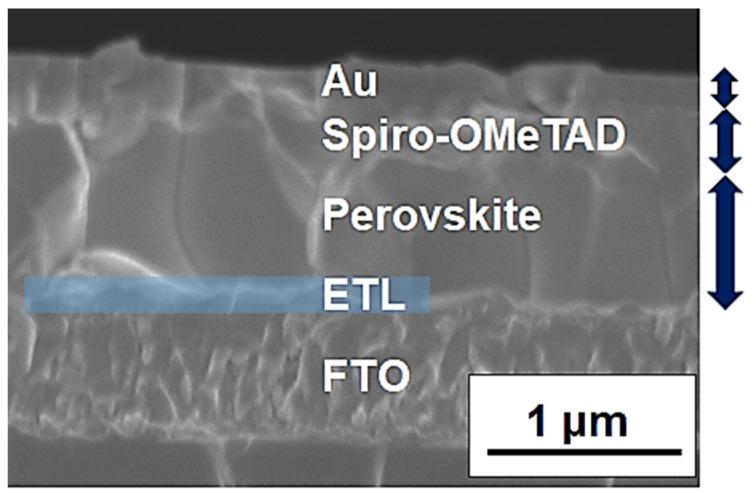
A cross-sectional SEM image of PSC using SnO_2_ particles formed at 80 °C.

A cross-sectional SEM image of the PSC, consisting of the 80 °C SnO_2_ sample, is presented in Figure 8. SnO_2_-based thin films were deposited on top of an FTO substrate using an ethanol-based nanoparticle solution combined with a TiO_2_ coating layer (see Materials and Methods section). We anticipated that the TiO_2_ layer could improve electron transport efficiency due to the cascade energy-level shift from the TiO_2_ to the SnO_2_ nanoparticle layer. This coating layer may also be effective in suppressing recombination at the surface of the smaller SnO_2_ nanoparticles. The thickness of the ETL section was found to be approximately 100~150 nm. The large grain size of the perovskite compound, characteristic of high-efficiency PSCs, was also confirmed in the image.

**Figure 9:**
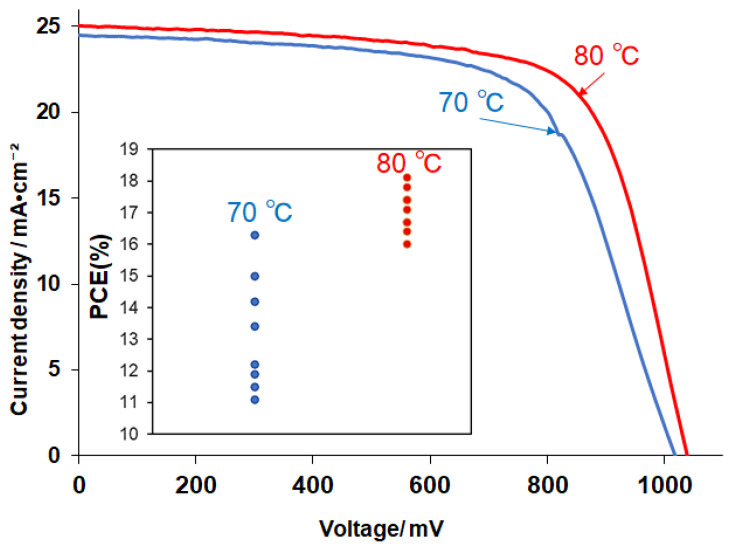
I-V curves of the best-performing devices using 70 °C and 80 °C SnO_2_ ETLs. The measurements were carried out under simulated full sunlight (100 mW/cm^2^). Insert is the distribution of PCE for both 70 °C and 80 °C devices.

Our primary focus was on the reaction-temperature-dependent performance of SnO_2_-based ETL in PSCs. To make a fair comparison, we evaluated two SnO_2_ devices that differed only in the SnO_2_ materials, as illustrated in Figure 9. In this figure, we present data for each of the best-performing devices, with 70 °C or 80 °C SnO_2_ as an ETL material. The 80 °C device produced a short-circuit current density (J_sc_) of 25.0 mA/cm^2^, an open-circuit voltage (V_oc_) of 1050 mV, a fill factor (FF) of 0.69, and a PCE of 18.1%, respectively. We found a marked improvement in efficiency for the 80 °C device compared to the 70 °C SnO_2_ sample: a J_sc_ of 22.5 mA/cm^2^, a V_oc_ of 1020 mV, an FF of 0.65, and a PCE of 16.2%. We also obtained a narrower distribution of the PCE for the 80 °C devices, in which 7~8 cells for each type were measured. The data indicated that the 80 °C devices showed a higher PCE and better reproducibility than the 70 °C devices. It appears likely that the self-assembly of small SnO_2_ nanoparticles, as observed using TEM for the 80 °C sample, is one of the key factors for achieving a high PCE. The relatively low V_oc_ and FF may have been due to defects in the perovskite layer [3], which was not optimized in this study.

## 4. Conclusions

In conclusion, the application of phenylalanine methyl ester hydrochloride (L) in our solution synthesis of SnO_2_ nanoparticles has substantial advantages for tuning the particle sizes to a single-nanometer scale as small as 3 nm in addition to promoting the hydrolysis and polycondensation of Sn(IV) ions to enhance the crystallinity of SnO_2_ samples. Our method of synthesis is unique in that a liquid catalyst is not required for employing the aromatic amino acid hydrochloride for the controlled crystal growth of SnO_2_, enabling us to develop a facile reaction system, which provides a new strategy for the precise size control of sub-10 nm SnO_2_ nanocrystals. As a result, due to efficient ligand interaction with Sn(IV) ions in an excess amount of the ligand ([L]/[Sn(IV)] = 20), tiny nanoparticles with a very narrow size distribution were formed. We then successfully produced SnO_2_ thin films on top of a conductive substrate via an environmentally friendly process using liquid precursors, relying only on ethanol for the dispersion. Ultimately, double-layered thin films consisting of the obtained nanocrystals (at the bottom) and a TiO_2_ coating layer (at the top) were developed to be used in perovskite solar cells (PSCs). It is thought that effective SnO_2_ synthesis with a hydrochloride salt along with reaction-temperature-dependent crystal growth and dispersion of the SnO_2_ nanocrystals, in which the SnO_2_ dispersion/assembly can be controlled by solely changing the reaction temperature, will prove useful in the design of high-performance SnO_2_ ETLs, furthering the development of solution-processed devices of not only PSCs but also dye-sensitized solar cells.

## Data Availability

Data are contained within the article.
